# Neuropeptides at the crossroad of fear and hunger: a special focus on neuropeptide Y

**DOI:** 10.1111/nyas.14179

**Published:** 2019-07-04

**Authors:** Lucas B. Comeras, Herbert Herzog, Ramon O. Tasan

**Affiliations:** ^1^ Department of Pharmacology Medical University Innsbruck Innsbruck Austria; ^2^ Neuroscience Division Garvan Institute of Medical Research Sydney New South Wales Australia

**Keywords:** neuropeptides, fear, anxiety, feeding, hunger, neuropeptide Y, NPY

## Abstract

Survival in a natural environment forces an individual into constantly adapting purposive behavior. Specified interoceptive neurons monitor metabolic and physiological balance and activate dedicated brain circuits to satisfy essential needs, such as hunger, thirst, thermoregulation, fear, or anxiety. Neuropeptides are multifaceted, central components within such life‐sustaining programs. For instance, nutritional depletion results in a drop in glucose levels, release of hormones, and activation of hypothalamic and brainstem neurons. These neurons, in turn, release several neuropeptides that increase food‐seeking behavior and promote food intake. Similarly, internal and external threats activate neuronal pathways of avoidance and defensive behavior. Interestingly, specific nuclei of the hypothalamus and extended amygdala are activated by both hunger and fear. Here, we introduce the relevant neuropeptides and describe their function in feeding and emotional‐affective behaviors. We further highlight specific pathways and microcircuits, where neuropeptides may interact to identify prevailing homeostatic needs and direct respective compensatory behaviors. A specific focus will be on neuropeptide Y, since it is known for its pivotal role in metabolic and emotional pathways. We hypothesize that the orexigenic and anorexigenic properties of specific neuropeptides are related to their ability to inhibit fear and anxiety.

## Graphical abstract

This review article discusses the hypothesis that specific neuropeptides released during states of hunger concomitantly reduce fear and anxiety. It highlights specific pathways and microcircuits where neuropeptides may interact to identify prevailing homeostatic needs and direct respective compensatory behaviors. A specific focus will be on neuropeptide Y, since it is known for its pivotal role in metabolic and emotional pathways.

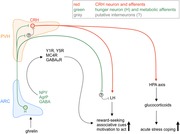

## Introduction

Frequently changing environmental conditions and the necessity to maintain an endogenous homeostatic balance force an individual into constantly adapting purposive behaviors.^1^ Genetically specified interoceptive neurons sense physiological needs and activate relevant brain areas to trigger physiological and behavioral responses with the aim to rebalance homeostatic equilibrium. Survival and reproduction are most commonly controlled by hunger, thirst, thermoregulation, and sexual repertoires. Furthermore, aggression, fear, and anxiety‐like behaviors support such essential needs and may assist in protecting the integrity of an organism from internal and external threats. For instance, during starvation, food intake and food seeking will eventually dominate over other life‐sustaining behaviors and encourage an individual to increase risk‐taking and foraging even in environments with acute dangers.^2^ On the other hand, in a state of satiety, safety desires may prevail and inhibit exploration of novel, putatively hazardous surroundings.^3^ Since a multitude of occasionally conflicting demands need to be addressed simultaneously, a thorough evaluation and identification of the most urgent needs may gate associated behavior accordingly. While the particular nature of individual survival circuits is increasingly well characterized, their mutual interaction and the decisive predominance of one over the others are still poorly understood. Evolutionarily conserved neuropeptide systems are ideally placed to control survival circuits across species, and dominate homeostatic behavior even in higher mammals and human beings.^4^ In this review, we will shed more light on the integrative role of neuropeptides in potential interaction pathways of individual survival circuits. We will focus specifically on the interplay of hunger and fear, two life‐sustaining forces that are in permanent competition, and we will highlight potential brain areas where dedicated neuropeptide systems may interact. Our main hypothesis is that specific neuropeptides, which are released during states of hunger, are concomitantly reducing fear and anxiety.

## Neuronal circuits of fear and anxiety

**Figure 1:**
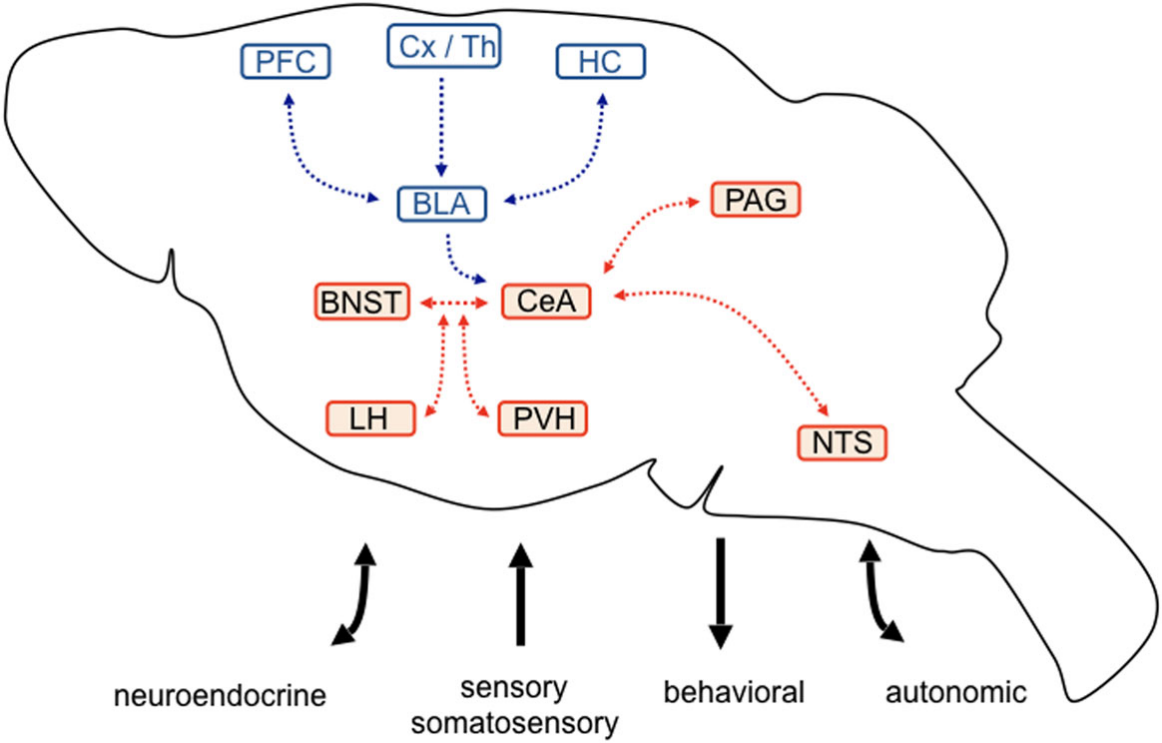
Proposed circuitry for fear and anxiety processing. Fear‐associated sensory and somatosensory stimuli are transmitted by cortical and thalamic (Cx/Th) inputs to the basolateral amygdala (BLA), which in turn integrates contextual and cognitive information by reciprocal connection with the hippocampus (HC) and prefrontal cortex (PFC) areas, respectively. Direct or indirect BLA projections communicate integrated information to the central amygdala (CeA), the major output nucleus of the amygdala. Intense crosstalk exists between the CeA and the bed nucleus of the stria terminalis (BNST), which is implicated in sustained fear and anxiety‐related behavior. Fear‐ and anxiety‐associated neuroendocrine, autonomic, and behavioral programs are organized by CeA and BNST projections toward the lateral (LH) and paraventricular hypothalamus (PVH), periaqueductal gray (PAG), and the nucleus of the solitary tract (NTS). Brain areas in red highlight potential interaction sites, where fear‐ and anxiety‐related stimuli may be integrated with metabolic information originating from peripheral autonomic, hormonal, or neuropeptidergic inputs.

Fear and anxiety are defensive states elicited by internal and external threats, with the overall aim of protecting an organism from potential harm.^5^ They are observed in multiple species and characterized by behavioral, autonomic, hormonal, and physiological reactions. Fear is considered a reaction toward an imminent, specific, or acute danger and involves learning and memory processes, while anxiety is largely inborn and a general reaction directed against a potential, more intricate and anticipated threat. However, fear and anxiety may also be seen as two behaviorally defined extremes of a continuum, since excessive, inappropriate, and chronic expression of both are hallmarks of anxiety disorders. A central hub for processing of fear memories is the amygdala complex.^6^ Fear acquisition appears to depend on the basolateral amygdala (BLA). Here, excitatory afferents from cortical and thalamic neurons convey auditory, visual, and somatosensory inputs of predictive cues that may coincide with the emergence of aversive stimulus–related signaling to form NMDA receptor–dependent synaptic plasticity.^7^, ^8^ The integrated information is transferred to the central amygdala (CeA), a main output node connecting to the hypothalamus, midbrain, and brainstem, to initiate corresponding endocrine, behavioral, and autonomic responses, respectively (Fig. 1). Furthermore, the bed nucleus of the stria terminalis (BNST), a rostral extension of the CeA, is also an intermediate processing point within the fear circuitry.^9^ It is important to note that the CeA is associated with an immediate and phasic fear reaction, whereas the BNST may be more relevant for sustained fear.^10^ However, both CeA and BNST receive considerable inputs from feeding‐related hypothalamic and brainstem nuclei and may thus integrate metabolic with fear‐related information (Fig. 1).

Fear extinction is generally considered as a new type of learning that interferes with the expression of established fear memories. Several lines of evidence highlight the importance of the BLA for extinction learning.^11^, ^12^ Reciprocal projections to the medial prefrontal cortex (mPFC) seem to define the relative contribution of fear and extinction.^13^ Furthermore, an extinction‐relevant amygdala microcircuit includes the intercalated neurons, a cluster of inhibitory GABA neurons that is located between the BLA and CeA^14^, ^15^ and facilitates feed‐forward inhibition of CeA output neurons.^16^ As a matter of fact, extinction memory is highly context specific and integrates hippocampal processes via long‐range projections connecting to the BLA, mPFC, and to a lesser extent, the CeA and BNST.

In contrast to fear, anxiety is characterized by approach‐avoidance behavior, but the underlying neurobiological correlates are less well defined.^5^ This may in part be due to its complex multifaceted combination of sustained arousal, high vigilance, and increased apprehension with specific defensive behaviors and autonomic reactions. Mounting evidence suggests a primary role of the BNST in anxiety‐related behavior, with different, in part opposing, functions depending on subnuclei and neuronal cell type.^17^

Thus, different microcircuits, cell types, and transmitter systems within defined brain areas induce a range of defensive behaviors comprising innate and sustained as well as learned and phasic responses. To identify how metabolic processes may shape fear and anxiety‐related behaviors, it may be useful to have a closer look at the primary entry points for the regulation of nutritional homeostasis and in particular on their direct projection targets within established fear and anxiety circuits.

## Neuronal circuits of homeostatic feeding

**Figure 2:**
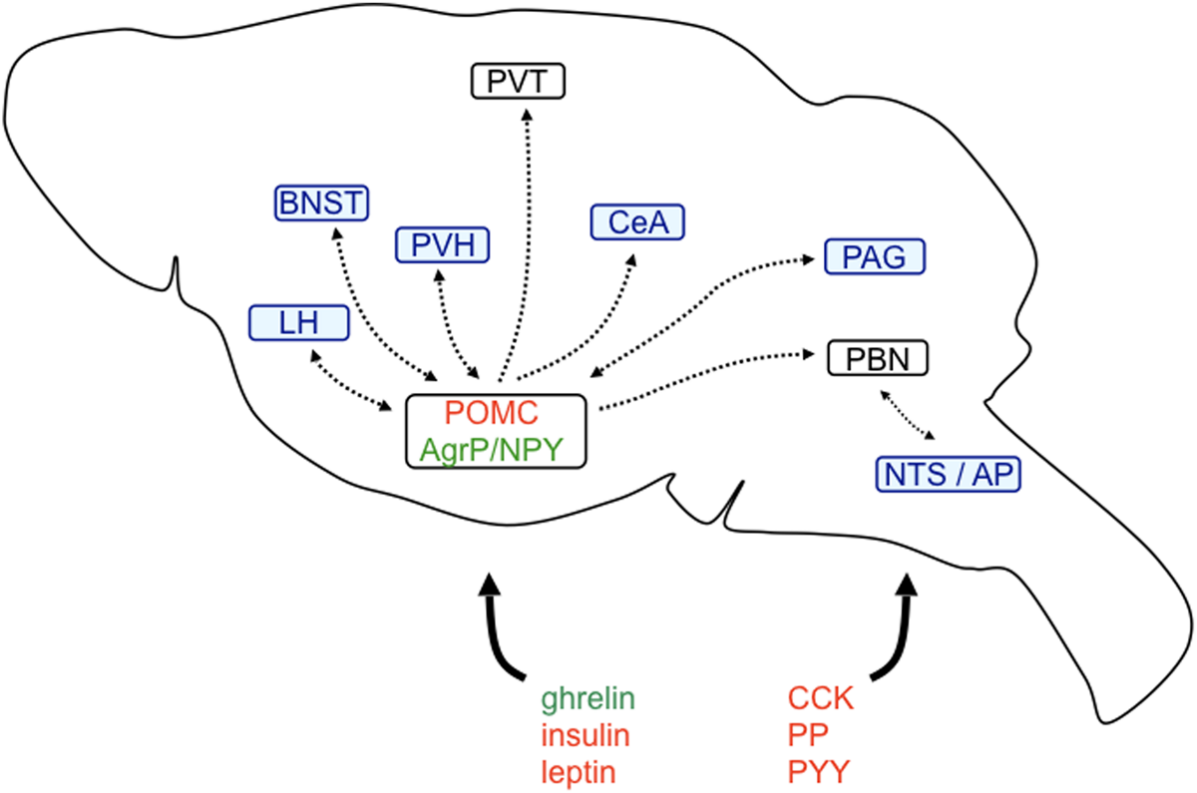
Fear‐relevant brain areas of the feeding circuitry. Ghrelin, leptin, insulin, cholecystokinin (CCK), peptide YY (PYY), and pancreatic polypeptide (PP) are released from the periphery and directly or indirectly activate/inhibit first‐order feeding neurons in the arcuate nucleus of the hypothalamus or in the nucleus of the solitary tract/area postrema (NTS/AP). First‐order arcuate orexigenic agouti‐related peptide/neuropeptide Y (AgRP/NPY) and anorexigenic proopiomelanocortin (POMC) neurons project to several second‐order feeding neurons throughout the brain. Highlighted in blue are those projection targets that may be relevant for the suppression of fear‐ and anxiety‐related behaviors. For more details, refer to the main text. BNST, bed nucleus of the stria terminalis; CeA, central amygdala; LH, lateral hypothalamus; PAG, periaqueductal gray; PBN, parabrachial nucleus; PVH, paraventricular nucleus of the hypothalamus; PVT, paraventricular nucleus of the thalamus.

**Figure 3:**
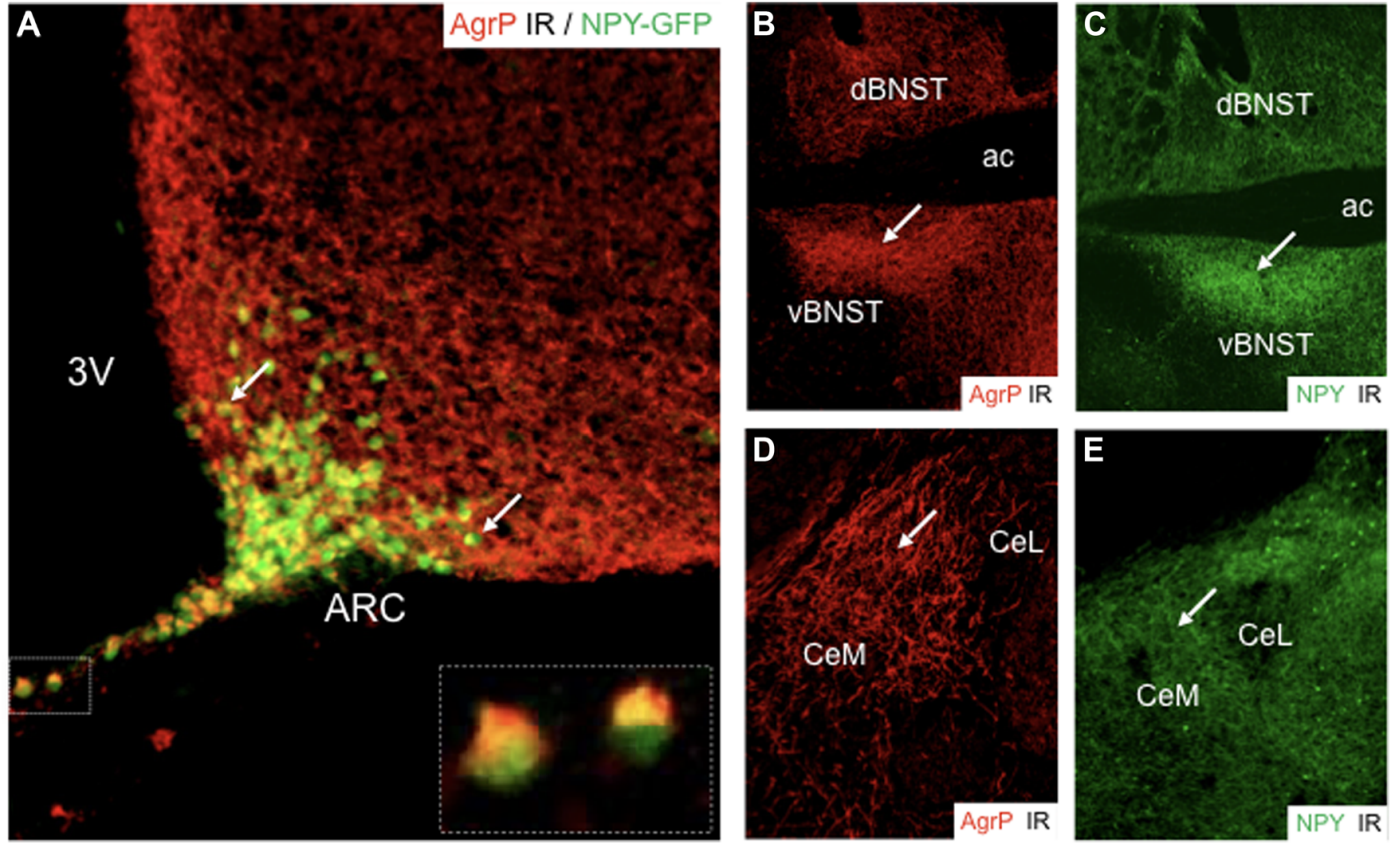
Immunohistochemical characterization of agouti‐related peptide (AgRP) and neuropeptide Y (NPY) staining in the arcuate nucleus of the hypothalamus (ARC), the bed nucleus of the stria terminalis (BNST), and the central amygdala (CeL, CeM). (A) Dual immunohistochemical staining of AgRP and NPY‐GFP demonstrates colocalization of AgRP and NPY in the ARC (arrows highlight examples of dual‐labeled neurons; insert depicts higher magnification of two neurons in the median eminence). Immunofluorescent staining of (B) AgRP and (C) NPY in the BNST; note the congruent distribution of the two neuropeptides and the particular enrichment in the ventral portion of the BNST (white arrows, vBNST). Immunofluorescent staining of (D) AgRP and (E) NPY in the central amygdala demonstrates strong expression of NPY in the medial subdivision (CeM) compared to the relative abundance of AgRP axons in the lateral portion (CeL). While AgRP labeling is derived exclusively from the ARC, NPY fibers may originate additionally from local sources (3V, third ventricle; ac, anterior commissure).

Regulation of feeding is maintained by the intense interaction of peripheral signaling molecules with the central nervous system (CNS). Ghrelin, a hormone released by enteroendocrine cells of the stomach, is increased upon fasting while suppressed by feeding. In contrast, cholecystokinin (CCK), insulin, glucagon‐like peptide‐1 (GLP‐1), leptin, pancreatic polypeptide (PP), and PYY originating from the gut and pancreas predominantly act as satiety factors and suppress food intake (Fig. 2).^18^ Nutritional states are monitored by a network of dedicated brain circuitries that integrate hormonal, vegetative, and nutrient signals. Such neuronal systems control food intake, food seeking, food hoarding, and other factors, such as consecutive fluid intake. In particular, neurons of the arcuate nucleus of the hypothalamus (ARC) and brainstem neurons of the area postrema (AP) and the nucleus of the solitary tract (NTS) serve as primary entry points for the control of food intake (Fig. 2). In the ARC, agouti‐related peptide and neuropeptide Y (AgRP/NPY)–containing neurons are in part accessible through a semipermeable blood–brain barrier (BBB) and sense peripheral signals of energy shortage, such as declining glucose levels or rising ghrelin levels (Fig. 3).^19^, ^20^ In contrast, adipocyte‐released leptin, PYY, and pancreatic insulin inhibit AgRP/NPY neurons.^21^ It is interesting to note that while acute activation of AgRP/NPY neurons stimulates food intake and foraging behavior independent of nutritional state, their chronic manipulation leads to desensitization (e.g., leptin resistance), suggesting adaptive compensatory systems.^1^ On the other hand, NPY levels do not adapt but rather increase with longer fasting durations.^22^ Nevertheless, projections of arcuate AgRP/NPY neurons to multiple brain areas contribute to the manifold behavioral facets of feeding (Fig. 2). For instance, the activation of AgRP/NPY neurons inhibits several neuronal subpopulations in the paraventricular hypothalamus (PVH) by the release of GABA and NPY, which results in increased food intake and associated metabolic and behavioral adaptations.^23^, ^24^ In the ARC, intermingled proopiomelanocortin (POMC) neurons are activated by PYY, leptin, GLP‐1, and insulin, leading to reduced food intake.^25^ AgRP/NPY neurons locally inhibit these POMC neurons. In addition, long‐range AgRP/NPY afferents to the parabrachial nucleus (PBN) counteract visceral malaise‐induced aphagia.^26^ It is well documented that AgRP/NPY neuron activation increases food intake, but this activity is rapidly suppressed when food consumption has started, highlighting their primary involvement in food‐seeking behavior.^27^ In this context, it is important to note that activation of AgRP/NPY neurons may generate a strong negative valence signal. Thus, mice avoid flavors and locations that were associated with extensive AgRP/NPY neuron activity, a phenomenon that drives them to keep on moving and search for food.^28^ It is actually food seeking, rather than food intake, that requires suppression of anxiety and fear, since foraging in potential dangerous environments may bear considerable risks. Along these lines, multiple, often redundant, arcuate projections modulate feeding‐precursory behaviors^29^ in particular, with various emphases and on different time scales, by the release of several neurotransmitters, many of which are evolutionarily conserved neuropeptides. Identification of these neuropeptides and how they interact with fear and anxiety circuits will be essential to find possible integrative nodes that could help to orchestrate conflicting survival behaviors.

## Relevance of neuropeptides for survival circuits

Neuropeptide transmitters are small peptides, some with distinct features and complex structures.^30^, ^31^ They predominantly act on G protein–coupled receptors, both in a stimulatory and inhibitory fashion. Neuropeptides are stored in large dense core vesicles and often released from extrasynaptic sites. In contrast to fast‐acting classical neurotransmitters that are reuptaken and synthesized in axon terminals, neuropeptides are translated from preexisting or newly transcribed mRNAs and transported along the axon to the release sites. One can assume that the great majority, if not all, neurons contain neuropeptide modulators in addition to fast‐acting classical transmitters. Since the cognate G protein–coupled receptors possess high affinities for neuropeptides, the latter may act on closely located sites at postsynaptic neurons, but could also travel longer distances by volume transmission,^32^ as exemplified by the anatomical mismatch of particular neuropeptides and their respective receptor distribution. For instance, AgRP/NPY neurons inhibit nearby POMC neurons by the release of NPY/ArgP and GABA. On the other hand, they are also targeted by long‐distance acting hormones, such as leptin, ghrelin, or insulin, that report about the prevailing homeostatic balance in the periphery.^1^ A similar mode of action was suggested within the brain, along the extracellular space or through the cerebrospinal fluid (CSF), following release into the ventricles.^33^ Thus, neuropeptides may act by several modes, thereby connecting pre‐ and postsynaptic neurons, distinct brain areas, or peripheral organs with the CNS. The integrative function of neuropeptides is apparently more modulatory than decisive, setting the stage for the efficacy of fast synaptic transmission. As a matter of fact, neuropeptides emerged as cornerstones of homeostatic balance by monitoring metabolic, reproductive, emotional‐affective, and motivational processes. While the physiological properties of neuropeptides are manifold, we will specifically focus here on those neuropeptides that target or are released by first‐order feeding neurons and thus either directly connect the periphery with the CNS or integrate metabolic signaling into wider brain networks. We will highlight their role in emotional‐affective behaviors and exemplify how they may mutually interact to drive purposive behaviors toward increased or decreased fear and anxiety, depending on the prevailing homeostatic condition.

## Neuropeptide Y

NPY belongs to a family of gut–brain peptides that also includes peptide YY (PYY) and PP.^34^, ^35^ In addition to hormonal functions by paracrine and endocrine release in the gastrointestinal tract, NPY also acts as a neurotransmitter in sympathetic ganglia and the CNS. Such diversity provides a pertinent framework for integrating peripheral homeostatic signals with central processes.^36^

NPY is expressed throughout the brain, with particularly high expression in limbic brain areas, such as the hippocampus, amygdala, and hypothalamus (Fig. 3).^37^, ^38^ The highest concentrations are present in neurons of the ARC, a central hub for energy homeostasis. In the ARC, NPY is predominantly colocalized with AgRP and GABA, and accounts for one of the strongest orexigenic stimuli. The other two members of the NPY family, PYY and PP, are absent from the CNS. They are predominantly expressed in L cells of the gut and in F cells of the pancreatic islets, respectively.^36^ Upon release, they may act either locally within the gastrointestinal tract and pancreas or may be secreted into the blood circulation and bind to Y‐receptors at distinct brain areas with an accessible BBB, such as the hypothalamus or AP.^39^

NPY modulates multiple physiological and pathophysiological processes, but it is best known for its orexigenic and anxiolytic properties.^19^, ^31^, ^40^, ^41^, ^42^ In mammals, at least five different G protein–coupled receptors (Y1R, Y2R, Y4R, Y5R, and y6R)^43^ have been cloned. While NPY and PYY display equally high affinities for all receptors, PP preferentially binds to Y4R, while the pharmacology of y6R is controversial.^44^, ^45^, ^46^ The latter receptor is present in mice and rabbits, but not in primates or rats. The cleavage products PYY_3‐36_ and NPY_3‐36_ retain binding affinity to Y2Rs and Y5Rs, whereas they loose their affinity for Y1Rs.^47^ Postsynaptic Y1R and presynaptic Y2R are the most abundant Y receptors in the CNS and are enriched in limbic brain areas.^48^

### The role of NPY in food intake

Peripheral hormonal signals of energy deficit and energy surfeit, such as ghrelin or leptin, activate or inhibit AgRP/NPY neurons of the ARC, respectively. AgRP/NPY neurons, in turn, project to a variety of brain areas, including PVH, PVT, BNST, PBN, or PAG, where they also corelease the inhibitory principal neurotransmitter GABA.^23^ While AgRP antagonizes melanocortin 4 receptor (MC4R), NPY inhibits arcuate POMC neurons and thyrotropin‐releasing hormone/MC4R neurons by acting on Y1/Y5R. A divergence of Y receptor activation by NPY has been observed, where Y5R activation results in increased food intake, while Y1R activation also increased food hoarding and foraging.^49^ On the other hand, stimulating Y2Rs^50^, ^51^ and Y4Rs by peripheral injection of PYY_3‐36_ and PP, respectively, resulted in a reduction of food intake in mice.^52^, ^53^, ^54^ The underlying mechanisms are still not entirely clear but may involve brainstem and hypothalamic circuitries. It is interesting to note that intracerebroventricular (ICV) injection of NPY increased food intake and spontaneous physical activity. At least the increase in locomotion seems to depend on Y2Rs and is an important prerequisite for food seeking.^55^ The central actions of Y2Rs on various feeding‐associated behaviors may differ from peripheral effects,^53^, ^56^, ^57^ and in particular the hunger‐ and fear‐modulating effects of Y2Rs in extrahypothalamic AgRP/NPY projection targets remain to be investigated.

### The role of NPY in fear and anxiety

Combining classical pharmacology and genetic manipulations with approach‐avoidance tests has demonstrated an anxiolytic role of NPY. These effects depend on the activation of postsynaptic Y1Rs in the amygdala, hippocampus, and brainstem.^58^ On the other hand, activation of presynaptic Y2Rs was predominantly anxiogenic.

Furthermore, various conditioning paradigms revealed that NPY is a strong suppressor of fear. Gutman *et al*.^59^ suggest that NPY reduces the expression of fear‐potentiated startle in the BLA. NPY also reduced fear expression during extinction and extinction recall, effects that are probably mediated by Y1Rs. However, it is important to mention that the fear‐suppressing and extinction‐promoting effects may also involve other Y‐receptors.^60^ For instance, local deletion of Y2Rs in the CeA increased the expression of cued fear, while overexpression of a Y2R‐activating agonist had the opposite effect.^61^ The underlying mechanisms are still elusive, but a memory‐modulating effect may be one potential contributing factor. Along this line, a recent study demonstrated that hippocampal Y2Rs reduced the expression of contextual fear and re‐established impaired fear extinction.^62^ A concomitant reduction of spatial memory suggests that NPY may reduce fear by a general interference with memory consolidation. These data are supported by two recent studies indicating that local Y2R activation in the BNST promoted fear extinction and reduced the reemergence of fear.^63^, ^64^ Furthermore, since fear and anxiety may engage different neuroanatomical pathways,^5^ the anxiogenic but fear‐suppressing properties of central Y2Rs may be explained in part by a different anatomical localization of the receptor.

Collectively, NPY unequivocally increases food intake and reduces fear and anxiety by activating Y1Rs. Regarding Y2Rs, both modulation of metabolic and emotional‐affective behavior were variables, in part also by segregating peripheral from central Y2R functions (Table 1). But how does NPY balance energy homeostasis with fear‐ and anxiety‐related behaviors and which other neuropeptides are directly or indirectly involved?

**Table 1:** Metabolically active neuropeptides and their role in fear and anxiety‐related behavior

Neuropeptide	Relevant release site	Feeding	Anxiety	Fear	Fear extinction
Neuropeptide Y (NPY)	Central	Increase: agonist at Y1R, Y5R	Decrease: agonist at Y1R, Y5R Increase: agonist at Y2R	Decrease: agonist at Y1R, Y2R	Facilitated: agonist at Y1R, Y2R
Agouti‐related peptide (AgRP)	Central	Increase: antagonist at MC4R	Decrease: antagonist at MC4R	Decrease (?)	No data
Melanocyte‐stimulating hormone (α‐MSH)	Central	Decrease: agonist at MC4R	Increase: agonist at MC4R	Increase: agonist at MC4R	No data
Corticotropin (CRH)	Central	Decrease: agonist at CRH2R	Increase: agonist at CRH1R	Increase: agonist at CRH1R	Impaired: agonist at CRH1R
Cholecystokinin (CCK)	Peripheral and central	Decrease: agonist at CCKAR	Increase: agonist at CCKBR	Increase: agonist at CCKBR	Impaired: agonist at CCKBR
Ghrelin	Peripheral	Increase: agonist at GHSR1a	Increase (?)	Increase (?)	Facilitated
Leptin	Peripheral	Decrease: agonist at LepRb	Decrease (?)	Decrease (?)	Facilitated (?)
Peptide YY (PYY)	Peripheral	Decrease: agonist at Y2R	No change	No data	No data
Pancreatic polypeptide (PP)	Peripheral	Decrease: agonist at Y4R	Variable	No change	Facilitated: agonist at Y4R

## Melanocyte‐stimulating hormone

The POMC gene encodes a prepropeptide that gives rise to a variety of mature signaling molecules, such as adrenocorticotropic hormone (ACTH), corticotropin‐like intermediate peptide, melanocyte stimulating hormones (α‐, β‐, and γ‐MSH), β‐endorphin, lipotropin (β‐ and γ‐LPH), and met‐enkephalin.^65^ In the CNS, POMC expression is restricted to the ARC and NTS, both of which are protected by the semipermeable BBB and thus readily accessible to peripheral signaling molecules.

### Role of α‐MSH in food intake

Functionally, chronic but not acute activation of POMC neurons in the ARC inhibits food intake, while ablation of arcuate POMC neurons results in increased food consumption and body weight.^66^ In particular, α‐MSH, which activates melanocortin receptors (MC3/4R) in the PVH, seems to be the main inducer of this anorexic effect.^67^, ^68^ It is interesting to note that POMC neurons in the NTS reduce food intake immediately upon acute activation, while food intake and body weight reduction upon chronic activation is short‐lived and followed by a rebound effect. This suggests separate mechanisms for immediate, short‐term satiety signaling originating from visceral afferents to NTS POMC neurons and long‐term suppression of food intake mediated by hormones targeting POMC neurons in the ARC. Projection targets of these hypothalamic POMC neurons overlap considerably with those of AgRP/NPY neurons (Fig. 3). Furthermore, while α‐MSH activates MC3/4R, AgRP is an endogenous antagonist or even inverse MC3/4R agonist, highlighting the opposing roles of POMC and AgRP/NPY neurons in food intake and related adaptive behaviors.^69^ One of the major questions is how this interaction may ultimately modify fear‐ and anxiety‐associated behaviors.

### Role of α‐MSH in fear and anxiety

Compared to the wealth of data that underscore an anxiolytic and fear‐suppressing role of NPY, evidence for an involvement of the melanocortin system is minimal. However, several studies suggest that the activation of MC3/4R is anxiogenic, while blockade reduces anxiety.^70^, ^71^, ^72^ For instance, antagonism of MC4Rs, either subcutaneously or orally, reduced anxiety under baseline conditions, following exposure to swim stress.^73^ Similarly, intranasal application of the MC4R antagonist HS014 decreased anxiety‐like behaviors following single prolonged stress.^74^ Since an important control, with intranasally applied MC4R antagonist but without stress exposure, was missing, the behavioral effects of HS014 treatment alone are not entirely clear. Furthermore, HS014 increased social interaction and reduced anxiety of isolated individuals to the level of group‐housed conspecifics.^70^ Activation of arcuate POMC neurons by stress exposure may be an underlying cause for reduced food intake and increased anxiety‐related behavior, both of which can be ameliorated by concomitant injection of an MC4R antagonist. Moreover, the medial amygdala seems to be crucial, since local application of α‐MSH increased anxiety‐like behavior while reducing food intake, suggesting another close relationship between metabolic and affective processes. On the other hand, while α‐MSH reversed IL‐1β–dependent fear memory impairment,^75^ injection of α‐MSH alone did not alter conditioned fear (Table 1). Data about an involvement of α‐MSH in fear extinction are not available yet.

## Corticotropin‐releasing factor and the hypothalamic–pituitary–adrenal axis

It is well known that stress alters feeding behavior, though the triggered response can be increased or decreased food intake. By initiating activation of the hypothalamic–pituitary–adrenal (HPA) axis, corticotropin‐releasing hormone (CRH) is a primary regulator of stress.^76^ Release of CRH from parvocellular neurons of the PVH stimulates the release of ACTH from the pituitary gland. ACTH, in turn, is released into the circulation and induces the secretion of cortisol/corticosterone from adrenal glands. Glucocorticoids prepare the body for a stress reaction and initiate the mobilization of energy from peripheral stores, while also exerting negative feedback regulation by activating glucocorticoid receptors in the hypothalamus. A characteristic attribute of CRH in the CNS is its low basal expression and its fast induction following emotional and metabolic challenge.

### Role of CRH in food intake

It is interesting to note that insulin‐producing pancreatic β cells depend on basal levels of glucocorticoids for survival, which are maintained by constitutive ACTH and CRH release. In addition, CRH is expressed in the hypothalamus and various other brain regions and may have local effects on homeostatic regulation as well, for instance, by inhibiting AgRP/NPY neurons in the ARC, comprising a direct and fast mechanism of how stress‐induced CRH release inhibits food intake. In the CeA, fasting/refeeding, acute social defeat stress, or lipopolysaccharide injections all activate local CRH neurons, indicating that CRH may indeed regulate stress and metabolic processes also outside hypothalamic areas.^77^ It is important to note that only fasting/refeeding, but not fasting alone, activated CeA–CRH neurons, contrasting with the effects of NPY, which is usually induced by fasting alone but not by refeeding. Opposite effects of CRH and NPY signaling within the amygdala in relation to stress and fear were already proposed earlier and may extend to food intake and energy homeostasis as well.^78^, ^79^ Indeed, CRH in the PVH can antagonize the NPY‐induced increase in food intake upon fasting,^80^ highlighting competing roles of these two neuropeptides in food intake. Collectively, these results reveal that CRH and the even more related urocortins inhibit food intake. This effect is likely mediated by the activation of CRH receptor 2 (CRHR2) and may be linked to fear or stress exposure. While CRHR1 is widely expressed in the CNS, CRHR2 is confined to specific brain areas, such as lateral septum, PVH, ventromedial hypothalamus, medial amygdala (MeA), and dorsal raphe.^76^ However, the exact role of CRH on food intake in these structures remains to be investigated.

### CRH in fear and anxiety

Ample evidence highlights the important role of CRH in adaption to fear, stress, and anxiety, which has been reviewed elsewhere.^76^ We will focus here on mechanisms that may specifically underscore the interaction of fear and food intake. Most striking are the diurnal peak secretion of CRH and its stress‐related rise. As such, fear triggers an immediate rise in CRH and consequent glucocorticoid release, which in turn regulates CRH levels to promote fear. Activation of CRHR1 in several limbic brain areas, including CeM, BNST, or PAG, results in increased anxiety and preferentially sustained fear reactions.^81^ These structures also receive projections from arcuate AgRP/NPY neurons, suggesting a possible interaction node with food intake.^29^, ^82^ While retention of conditioned fear may be enhanced by CeA–CRH neurons,^83^ the overall role of CRH in the CeA is still controversial. On the other hand, retention of fear extinction was impaired by CRH, but facilitated by CRHR1 antagonists.^84^ In particular, the chronic elevation of CRH promotes sustained fear behaviors, likely in the BNST. These data put CRH in a pivotal position to inhibit appetite and respond to aversive stimuli by modulating multiple brain areas and orchestrating a variety of automated, protective behaviors (Table 1).

## Ghrelin

Ghrelin is synthesized in the stomach and is the endogenous ligand for the G protein–coupled growth hormone secretagogue receptor (GHSR1a).^85^ In the brain, GHSR1a is expressed predominantly in the anterior pituitary gland and ARC, but also in the hippocampus, substantia nigra, ventral tegmental area (VTA), and raphe nuclei.^86^, ^87^ Ghrelin reaches its targets by the blood circulation and directly interacts with receptors in brain areas with a permeable capillary zone, such as in the ARC,^88^ or may travel to brain areas without direct access to the bloodstream through the CSF.^89^

### Role of ghrelin and food intake

Ghrelin induces food intake by modifying hypothalamic activity. ICV and intraperitoneal (IP) administration of ghrelin in freely feeding rats stimulated food intake, plasma growth hormone concentration, and release of ACTH.^90^

In the ARC, the action of ghrelin is mediated by orexigenic AgRP/NPY neurons,^88^ with almost all AgRP/NPY neurons expressing GHSR1a. Furthermore, intra‐PVH injection of ghrelin increased food intake, while siRNA‐mediated knockdown of GHSR1a reduced body weight and blood ghrelin levels.^91^, ^92^ The PVH can also directly modulate the activity of the ARC, sending excitatory projections to arcuate AgRP/NPY neurons to induce feeding,^93^ indicating a feedback circuit between ARC and PVH to control different aspects of feeding and body weight regulation.

Interestingly, ablation of ghrelin‐producing cells in adults did not modify food intake, body weight, or resistance to a high‐fat diet, even with an almost complete reduction of ghrelin plasma levels, suggesting compensatory, redundant regulation of food intake.^94^

### Role of ghrelin in fear and anxiety

**Figure 4:**
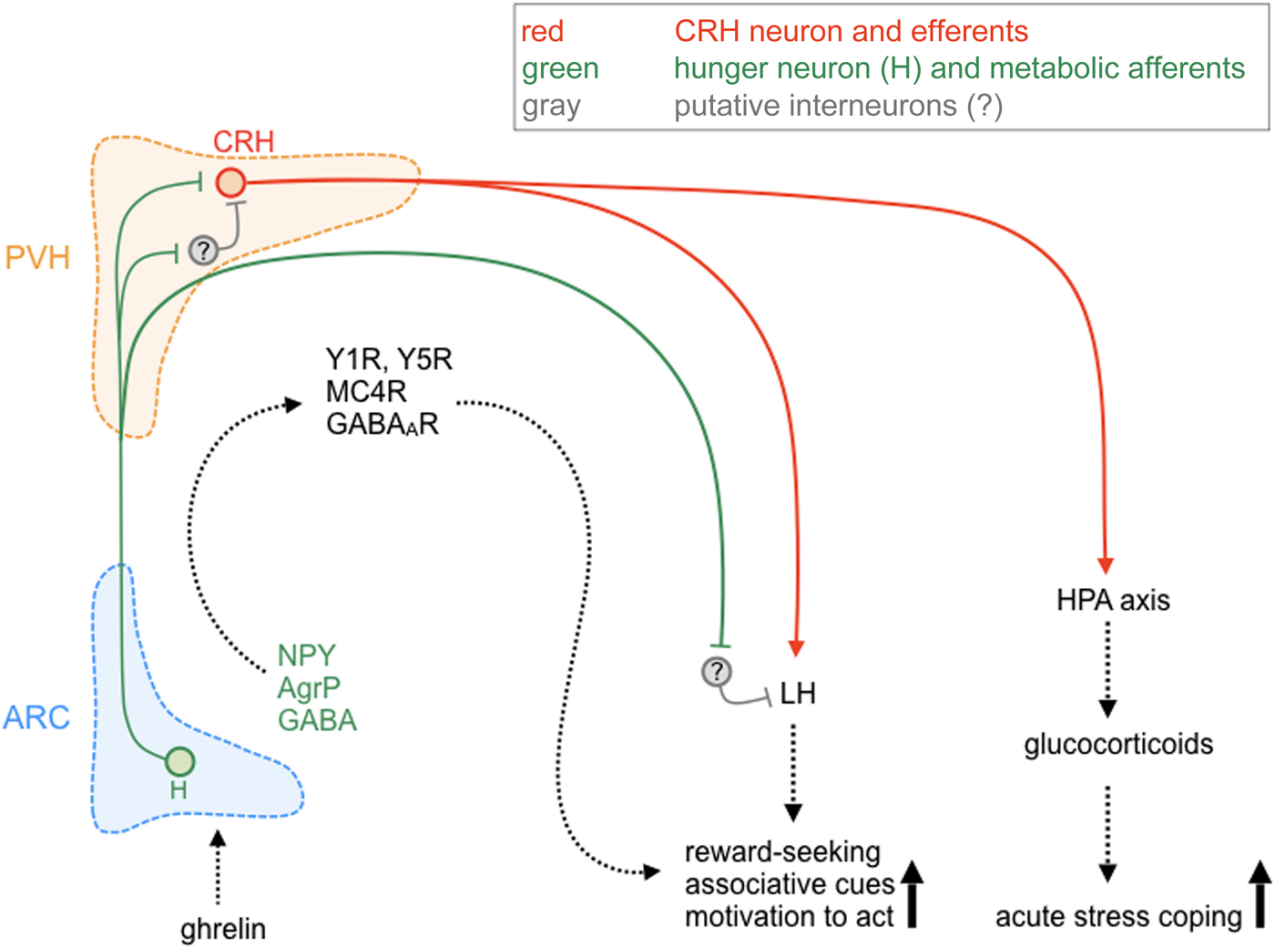
Proposed interaction of orexigenic agouti‐related peptide/neuropeptide Y (AgRP/NPY) neurons with behaviorally relevant neurons of the paraventricular (PVH) and lateral hypothalamus (LH). Hunger may control emotional‐affective processes through the activation of the hypothalamic–pituitary–adrenal (HPA) axis and by adapting associative and reward‐seeking behaviors. Upon hunger, the stomach releases ghrelin, which drives the activation of AgRP/NPY neurons (green) of the arcuate nucleus of the hypothalamus (ARC). AgRP/NPY neurons project to the PVH, where they activate corticotropin‐releasing hormone (CRH)–positive neurons (red). Since NPY and AgRP are inhibitory neuropeptides, this activation could be mediated by a disinhibitory interneuron (gray). Ghrelin could also directly modify the activity of the PVH. Continuous release of CRH and subsequent activation of the HPA axis initiates an acute stress response and could also activate the lateral hypothalamus (LH), which integrates multiple afferents from the cortex, amygdala, and forebrain areas to convert motivated, reward‐seeking, and associative stimuli into adaptive behavior.

Several studies^95^, ^96^, ^97^, ^98^ illustrate that ghrelin induces anxiety‐like behaviors, while injection of a CRHR antagonist blocked this effect, suggesting an interaction of CRH and ghrelin.^98^ CRH‐releasing neurons in the PVH initiate the activation of the HPA axis responsible for stress adaptation by corticosterone release (Fig. 4). ICV injection of ghrelin stimulates ACTH cell hypertrophy and proliferation, and promotes ACTH and corticosterone release.^99^

Using *in situ* hybridization, low GHSR1a mRNA abundance was detected in the PVH.^86^, ^87^ In addition, peripheral, ICV, or intra‐PVH administration of ghrelin activated c‐Fos in CRH‐producing neurons and induced CRH expression.^92^ However, since CRH neurons do not express GHSR1a mRNA, their activation may be through an indirect mechanism.

While initial investigations did not detect GHSR1a mRNA in the amygdala,^86^, ^87^ a recent study described GHSR1a expression in the lateral amygdala.^100^ Moreover, intra‐amygdala injections of ghrelin enhanced passive avoidance learning^101^ and improved memory retention in mice,^102^ indicating that at least exogenous ghrelin can directly modify amygdala activity. Importantly, fasted mice exhibited facilitated fear extinction, which was accompanied by increased ghrelin plasma levels,^2^, ^103^ while blocking of GHSR1a reversed this effect (Table 1).

## Leptin

Leptin is produced by white adipocyte tissue^104^ and is highly correlated with white fat energy stores.^105^ Leptin levels are elevated in fed animals, but are reduced upon fasting.^106^ The leptin receptor (LepRb) is a type I cytokine receptor coupled to tyrosine kinase.^107^ LepRb‐mutant mice are characterized by hyperphagia‐induced obesity, hyperlipidemia, and insulin resistance.^108^ In the brain, LepRb expression was reported in the cerebral cortex, hippocampus, thalamus, and in particular in the hypothalamus.^109^

### The role of leptin in food intake

Leptin acts as an indicator of fat reserves, modulates energy expenditure, and reduces food intake also by ICV injection.^109^ LepRb is expressed by arcuate AgRP/NPY and POMC neurons, but producing opposite effects upon activation. Leptin upregulates, whereas food deprivation decreases, the levels of POMC mRNA.^110^ On the other hand, ICV injection of leptin decreased arcuate NPY mRNA levels.^111^ In addition, leptin directly depolarized POMC neurons and reduced inhibition of POMC neurons by NPY.^112^ Thus, in the absence of NPY, *Lep* ***^ob^*** ^/^ ***^ob^*** mice displayed reduced body weight and food intake, suggesting that NPY is a central effector of leptin action. Leptin also increased mRNA levels of CRH^111^ and vasopressin in the PVH of rats, and may thus organize metabolic and emotional‐affective processes.

### Leptin in fear and anxiety

Only little is known about leptin's role in fear and anxiety. Leptin‐deficient mice displayed increased anxiety‐related behavior in the light–dark test.^113^ In addition, mice with LepRb selectively knocked out in dopamine neurons exhibited an anxiogenic‐like phenotype in approach‐avoidance testing.^114^, ^115^ Regarding fear, both systemic and intra‐amygdala injections of leptin impaired fear expression when injected before recall (Table 1).^116^

## Cholecystokinin

CCK is synthesized by neuroendocrine cells of the proximal intestinal mucosa^117^ and secreted in the presence of luminal fat and protein. In addition, a considerable amount of CCK is synthesized by CNS neurons.^118^ However, CCK in plasma is almost exclusively derived from the intestinal mucosa. There are several variants of CCK produced from the common precursor pro‐CCK and all of them act as ligands for CCK receptors. In the gut, higher molecular weight forms, like CCK58 and CCK33, are predominant, whereas in the brain the smaller, sulfated CCK8 is the prevalent molecular variant.

Two CCK receptors, CCKAR and CCKBR, with about 50% homology have been cloned. Both are G protein–coupled receptors that activate a large number of transduction pathways, ultimately leading to altered gene expression.^119^ CCKBR is activated by multiple CCK variants and pentagastrin. However, the activation of CCKAR requires an amino‐terminal extension with sulfated tyrosine only present in CCK. CCK receptors are widely expressed throughout the entire brain, with some areas, such as hypothalamus, expressing mainly CCKBR, while others, such as the amygdala, mainly express CCKAR.^120^

### Role of CCK in food intake

CCK plasma levels increase rapidly after food consumption,^121^ while food deprivation decreases intestinal CCK mRNA.^122^ CCK acts therefore as a satiety signal and repeated injections of CCK reduce meal size but increase meal frequency^123^ in rats and humans alike.^124^ In particular, antagonists of CCKAR blocked the satiety effects of exogenous CCK and increased meal size,^123^ identifying CCKAR as the main inducer of anorexigenic effects.

Despite its effects on food intake, IP injection of CCK does not activate arcuate neurons. However, CCK increases the number of c‐Fos–immunoreactive neurons in various other brain areas, such as NTS, PVH, AP, supraoptic nucleus, and CeA,^125^ and its anorexigenic effects seem to be dependent on NTS activation.^126^ Since gastric vagotomy blocks the satiety effects of CCK,^127^ it is generally assumed that CCK shifts the balance of how the vagal nerve responds to orexigenic and anorexigenic stimuli. Furthermore, it was suggested that CCK inhibits the appetite‐stimulating effects of peripheral ghrelin,^128^ activates the release of anorexigenic acting CRH,^129^ and may suppress arcuate NPY. In addition, CCK acts as a neurotransmitter in the CNS and may reduce food intake by the activation of central CCKARs, a phenomenon highly relevant for integrating metabolic balance with fear and anxiety‐related behaviors.^130^

### The role of CCK in fear and anxiety

Excellent reviews on the role of CCK in fear on anxiety have been published elsewhere.^130^, ^131^ In brief, CCK agonists increased anxiety‐like behavior in several behavioral paradigms.^132^ These effects were attenuated by ICV injections of NPY and potentiated by the Y1R antagonist BIBP3226,^133^ suggesting an interaction of anorexigenic CCK and orexigenic NPY also in emotional‐affective processing. In several human studies, CCK was reported to induce panic and fear.^134^ Interestingly, ICV injections of CCKBR antagonists blocked CCK‐induced anxiety, whereas antagonists of CCKAR did not,^135^ illustrating that the anxiogenic effects of CCK are mediated by CCKBR receptors. The effects of CCK in anxiety could be a consequence of its activation of the HPA axis. However, rats with a high‐anxiety phenotype displayed increased expression of CCKBR in the BLA,^136^ and intra‐BLA injections of a CCKBR agonist increased acoustic startle responses.^137^ In fear‐conditioning paradigms, IP injections of CCKBR antagonists blocked fear expression and facilitated extinction.^138^ Collectively, CCK increases anxiety and fear‐related behaviors and can even induce panic attacks (Table 1).

## Possible interactions in the brainstem and hypothalamus

In the hypothalamus and brainstem, first‐order neurons mediate immediate responses to metabolic stimuli. Ghrelin and leptin increase and decrease food intake, respectively, mainly by modulating arcuate AgRP/NPY and POMC neurons. Interestingly, acute fasting increased levels of ghrelin and promoted the extinction of conditioned fear.^103^ While GHSR1a receptors in the ARC and NTS/AP are readily accessible to circulating ghrelin, GHSR1a receptors in the amygdala and other brain areas are not.^87^ Intrinsic receptor activity and exogenous application of ghrelin may in part explain the observed effects. On the other hand, PVH neurons are also only partially protected by a semipermeable capillary zone and intra‐PVH injections of ghrelin produced orexigenic effects,^97^ modified the activity of CRH neurons,^139^ and triggered the activation of the HPA axis and the release of corticosterone. Several reports suggest that glucocorticoids promote fear extinction,^140^ highlighting a possible interaction pathway between hunger and fear. Furthermore, PVH CRH neurons interact with the lateral hypothalamus,^141^ a brain area that integrates metabolic with reward, emotional, and cognitive cues^142^ (Fig. 4). In particular, its ability to transition from motivation to action selection and response initiation is a prerequisite for reward seeking. Thus, hunger results in a well‐balanced activation/inhibition of diverse subpopulations in the paraventricular and lateral hypothalamus to generate purposive feeding‐related behaviors.

In a similar fashion, leptin released from adipocytes must enter brain parenchyma to target its cognate receptors. Leptin accumulates in the choroid plexus and enters the ARC via the median eminence, where it regulates food intake.^143^, ^144^ High expression of LepRb along endothelial cells suggests an active transport mechanism into the brain. LepRb seems to be especially involved in feeding‐associated, rewarding behaviors, but not in food intake per se.^144^ Whether they are also a link to fear and anxiety remains open.

IP injection of CCK reduces food intake, probably by modulating vagal afferents to the NTS. In addition, peripheral injected CCK potentiated anxiety‐induced 5‐HT release in the mPFC in guinea pigs.^145^ This is an interesting finding, since both CCK and 5‐HT are known to reduce food intake and promote anxiety‐related behaviors.^130^ It also demonstrates that peripherally released CCK does not only modulate food‐intake, but also emotional‐affective behaviors. CCK‐containing first‐order neurons of the NTS project to the PBN, which is involved in anorexigenic activity due to visceral malaise,^146^ but may also mediate panic‐induced increase in respiration.^147^ A dense network of CCK‐containing anxiogenic and fear‐promoting pathways exists in the brain,^130^ which is particularly elaborated in cortical areas, BLA, and hippocampus. Thus, peripheral CCK may activate an assembly of anorexigenic brainstem and hypothalamic nuclei, which in turn trigger the activation of higher order CCK clusters in the cortical–amygdala–hippocampal structures to simultaneously increase fear‐ and anxiety‐related behaviors and suppress fear extinction. Such a CCK network, although still a more hypothetical concept, would be useful in coordinating peripheral energy supplies with adaptive behavioral programs by a single neuropeptide.

Anatomically, the ARC serves as a primary entry point for the modulation of food intake, and its projection targets are possible integrating nodes for fear‐ and anxiety‐related stimuli.^29^, ^148^ In a Pavlovian food challenge task, optogenetic activation of AgRP/NPY neurons increased the time in a previously shocked chamber, in the presence and absence of direct food access, suggesting higher risk taking and/or reduced fear.^3^ In addition, anxiolysis was demonstrated by increased open arm time on the elevated plus maze and was reminiscent of fasted mice. Motivational state competition following the activation of AgRP/NPY neurons could be instrumental for suppressing fear and anxiety in the presence of food,^3^ conceivably by those AgRP/NPY projections directly targeting BNST, CeM, and PVT^148^ (Fig. 2).

## Possible interactions in the CeA

The amygdala complex is fundamental for fear and anxiety, but understanding of the significance of orexigenic and anorexigenic neuropeptide transmitters within different amygdala nuclei is still evolving. Inhibition of fear memory consolidation and facilitation of fear extinction in fasted mice was associated with altered c‐Fos expression in intercalated neurons,^2^ a subpopulation of inhibitory amygdala neurons connecting the BLA with the CeA. In particular, facilitation of feed‐forward inhibition in this amygdala microcircuit is the most relevant for fear extinction^16^ and was also increased in fasted mice.^2^ Thus, upon food deprivation, NPY derived from AgRP/NPY neurons may suppress fear and promote extinction in the amygdala by supporting a microcircuit that inhibits CeM activity. This would directly link food intake with emotional‐affective behaviors.

**Figure 5:**
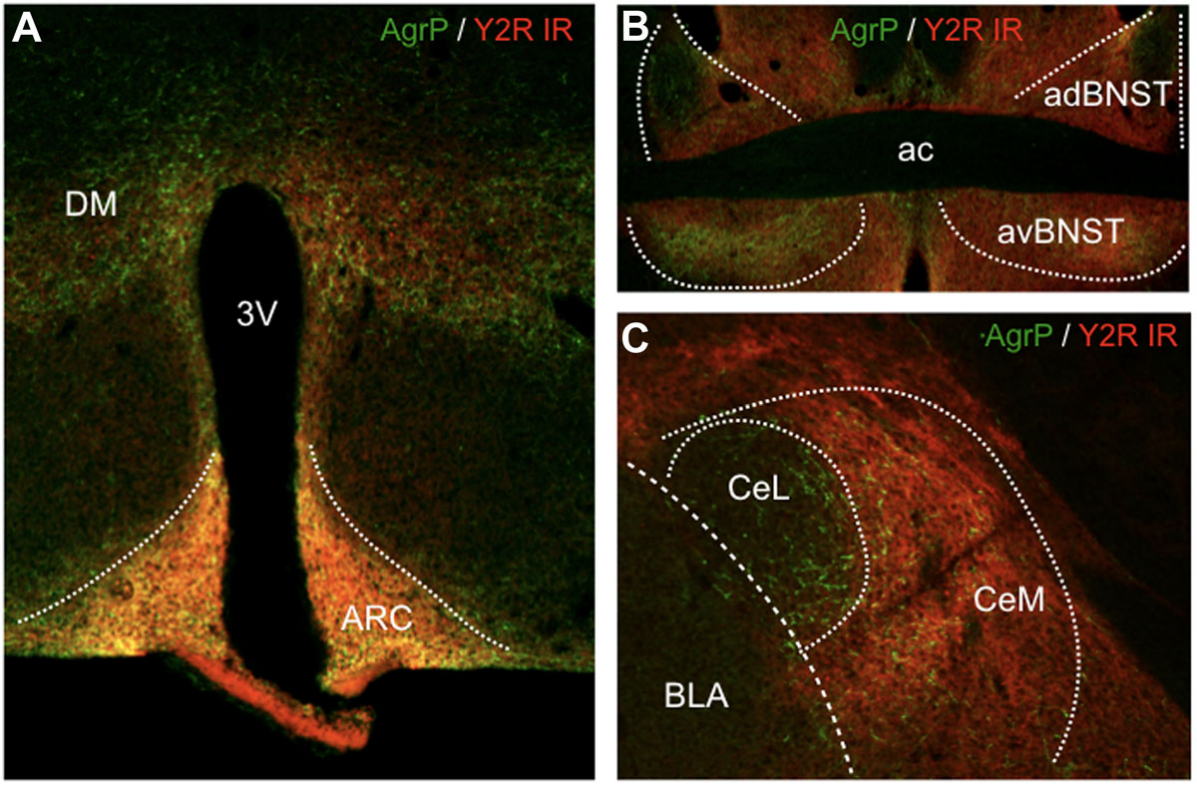
Dual immunofluorescent staining for agouti‐related peptide (AgRP) and neuropeptide Y Y2 receptor (Y2R). (A) Dual immunofluorescent staining for AgRP (green) and Y2R (red) in the hypothalamus demonstrates considerable colocalization within the arcuate (ARC) and dorsal nucleus (DM). (B) Extensive overlap of AgRP (green) and Y2R (red) immunoreactivity (IR) in the ventral aspect of the bed nucleus of the stria terminalis (vBNST). (C) Overlapping distribution of AgRP and Y2R IR in the CeM, whereas in the CeL only AgRP but no Y2R labeling was detected. Note that the overlapping distribution is within the same brain areas, but not in the same fibers, suggesting that NPY released from AgRP/NPY terminals may activate presynaptic heteroreceptors of different neurons.

The CeM is the major output nucleus of the amygdala and as such a central hub for the control of fear expression. Mounting evidence indicates that it is not only a passive relay station, but rather a site of intense plasticity, integrating a variety of different input modalities to initiate an adapted behavioral response.^149^ CeM neurons contain Y2Rs,^150^ which are functionally expressed throughout the axonal projections to the BNST^151^ and may inhibit GABA release (Fig. 5). Pharmacological and genetic studies have in particular highlighted amygdalar Y1 and Y2Rs as the predominant mediators of fear suppression,^42^, ^58^ but whether NPY is released by local interneurons or also from axon terminals originating from other brain areas, such as the hypothalamus and brainstem, is still unclear.

**Figure 6:**
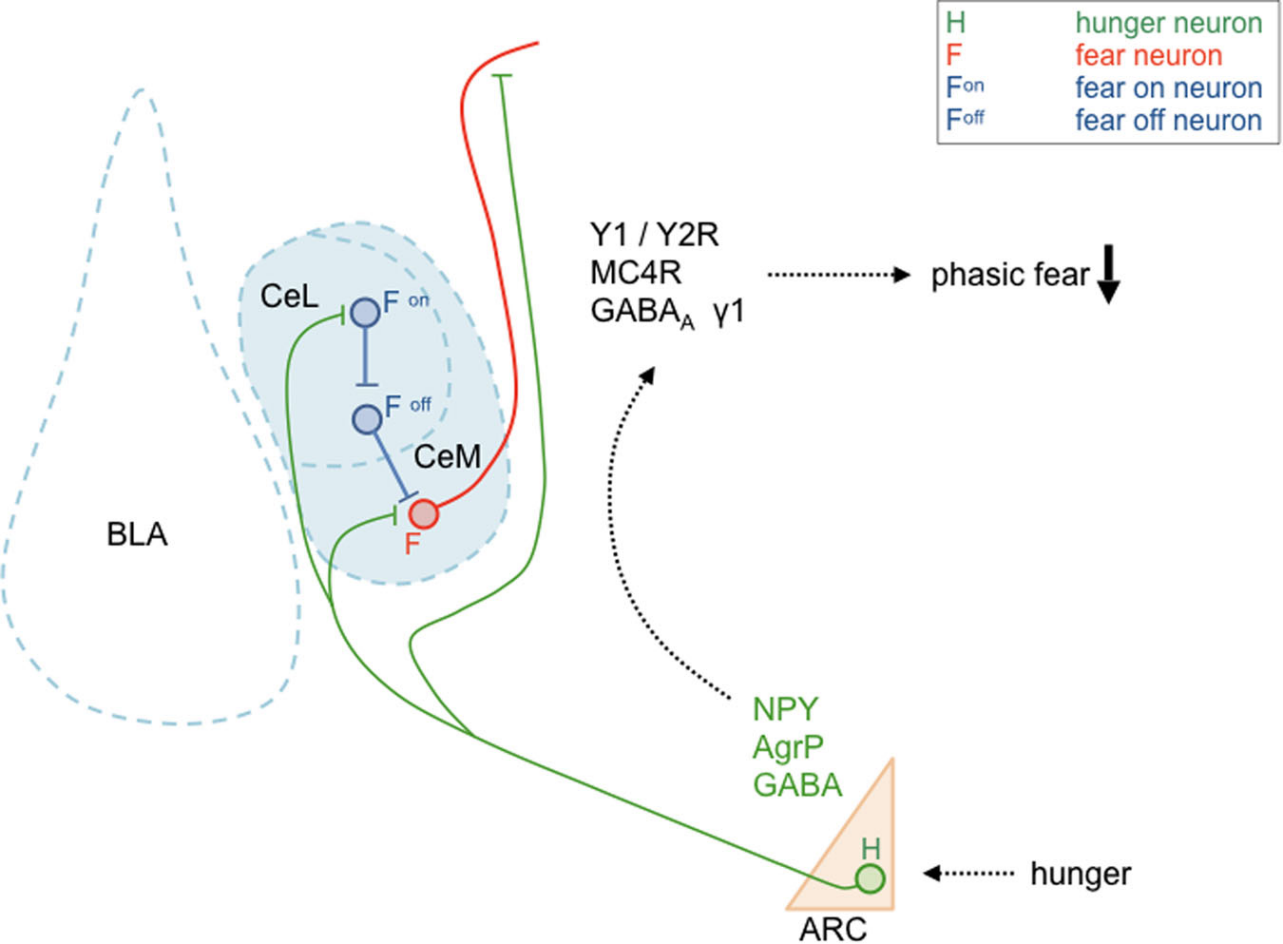
Proposed microcircuit of agouti‐related peptide/neuropeptide Y (AgRP/NPY) axons and several central amygdala subpopulations. Hunger activates arcuate AgRP/NPY neurons that project to the central amygdala. There, centromedial (CeM) fear neurons may be inhibited by the activation of Y2 receptor (Y2R) and GABA_A_ receptor (GABA_A_ R), and by antagonism of melanocortin 4 receptor (MC4R), within the CeM and/or along the axonal efferents of CeM neurons. Furthermore, inhibition of centrolateral (CeL) fear neurons (F^on^) may disinhibit fear off (F^off^) neurons, which in turn inhibit CeM fear neurons. The overall effect will be a reduction of phasic fear during hunger.

The amygdala is a rich source of NPY, and AgRP/NPY fibers from the ARC project to the CeA, MeA, and BNST^82^ (Fig. 5), but their role in behavioral changes that are related to and supportive of homeostatic feeding is only beginning to emerge. It is interesting to note that AgRP/NPY fibers are not only present in the CeM, but also in the BNST and all along the Y2R‐containing axonal bundles that connect both structures (Fig. 5). Thus, during hunger, ArgP/NPY neurons may strongly suppress CeM output by the activation of Y2R‐containing fibers (Fig. 6).

It is interesting to note that both local deletion of Y2Rs and local overexpression of a Y2R‐specific agonist resulted in corresponding changes in the CeM and BNST, respectively, supporting the idea of an intensely interconnected structure.^61^, ^152^ Such a concept would start with increased ghrelin release upon short‐term fasting, activating AgRP/NPY neurons, which in turn target PVH neurons to increase food intake, while parallel projections to the amygdala could suppress fear expression by the activation of Y2Rs via an interconnected network of CeM–BNST neurons (Fig. 6). However, NPY is not the only neurotransmitter released from orexigenic AgRP/NPY neurons. In particular, AgRP may serve as an endogenous antagonist of central MC4R, enabling synergistic effects of Y receptor activation and MC4R blockade in reducing anxiety‐related behaviors.^153^ In the CeA, activation of MC4R results in inhibition of N‐type calcium channels, reducing GABA release.^154^ Furthermore, pharmacological stimulation of MC4R resulted in intense activation of the amygdala, while antagonism of N‐type calcium channels led to a specific activation of the CeL subdivision. This is also supported by increased c‐Fos immunohistochemical staining in the CeL following AgRP infusion.^155^ It is generally assumed that specific inhibitory projections from CeL to CeM reduce fear expression by inhibiting CeM output neurons^156^ (Fig. 6). Thus, it is tempting to speculate that inhibition of the CeA MC4Rs by AgRP shifts activity of the CeL in a subpopulation‐specific manner toward inhibition of CeM output neurons and a reduction of fear expression, while favoring extinction learning (Fig. 6). Along these lines, central or intranasal applications of MC4R antagonists have shown promising results in reducing various behavioral symptoms associated with post‐traumatic stress disorder.^73^, ^74^, ^157^ In addition to neuropeptides, GABA released from AgRP/NPY neurons may mediate tonic inhibition of anxiogenic CRH release in the CeL.^158^ Indeed, in high anxiety–related behavior mice, α5 and γ1 subunits of the GABA_A_ receptor were specifically downregulated, a phenomenon that may reduce tonic inhibition and increase anxiety.^159^

Fear‐potentiating CeL afferents onto somatostatin neurons originate also from the PVT,^160^ another target of AgRP/NPY neurons. Activation of AgRP/NPY axon terminals in the PVT induced food intake only upon strong, probably nonphysiological stimulation,^29^ indicative that feeding is not the primary function of these projections. However, all three transmitters of ArgP/NPY neurons (AgRP, NPY, and GABA) may induce inhibition of the PVT and thus also inhibit the potentiation of conditioned fear. Considering the PVT–CeL–CeM–BNST as a central executive crossroad of fear development, arcuate AgRP/NPY neurons are ideally suited to inhibit this pathway at several neuralgic points and with various neurotransmitters, wherein GABA may be fast‐acting, while the respective neuropeptides could synergistically alter tonic signaling on a longer timescale. During states of increasing hunger and continuous homeostatic imbalance, AgRP/NPY neurons may inhibit passive freezing behavior and shift the equilibrium toward an active coping strategy, enabling an organism to adapt to the prevailing condition and search for food even in an environment or time window of increased threats. It was recently proposed that AgRP/NPY neuron activation generates a negative valence that urges the animal to move on and search for food.^28^ Along this line, activation of CRH neurons in the CeA may facilitate active responses toward external threats and suppress passive freezing behavior.^161^ Thus, the fear‐suppressing and extinction‐promoting effects of short‐term fasting, seen in *Drosophila*, mouse, and healthy human subjects,^2^, ^103^, ^162^, ^163^, ^164^ may be at least in part mediated by activation of specific CeL subpopulations that favors an active coping strategy (Fig. 6).

## Possible interactions in the BNST

**Figure 7:**
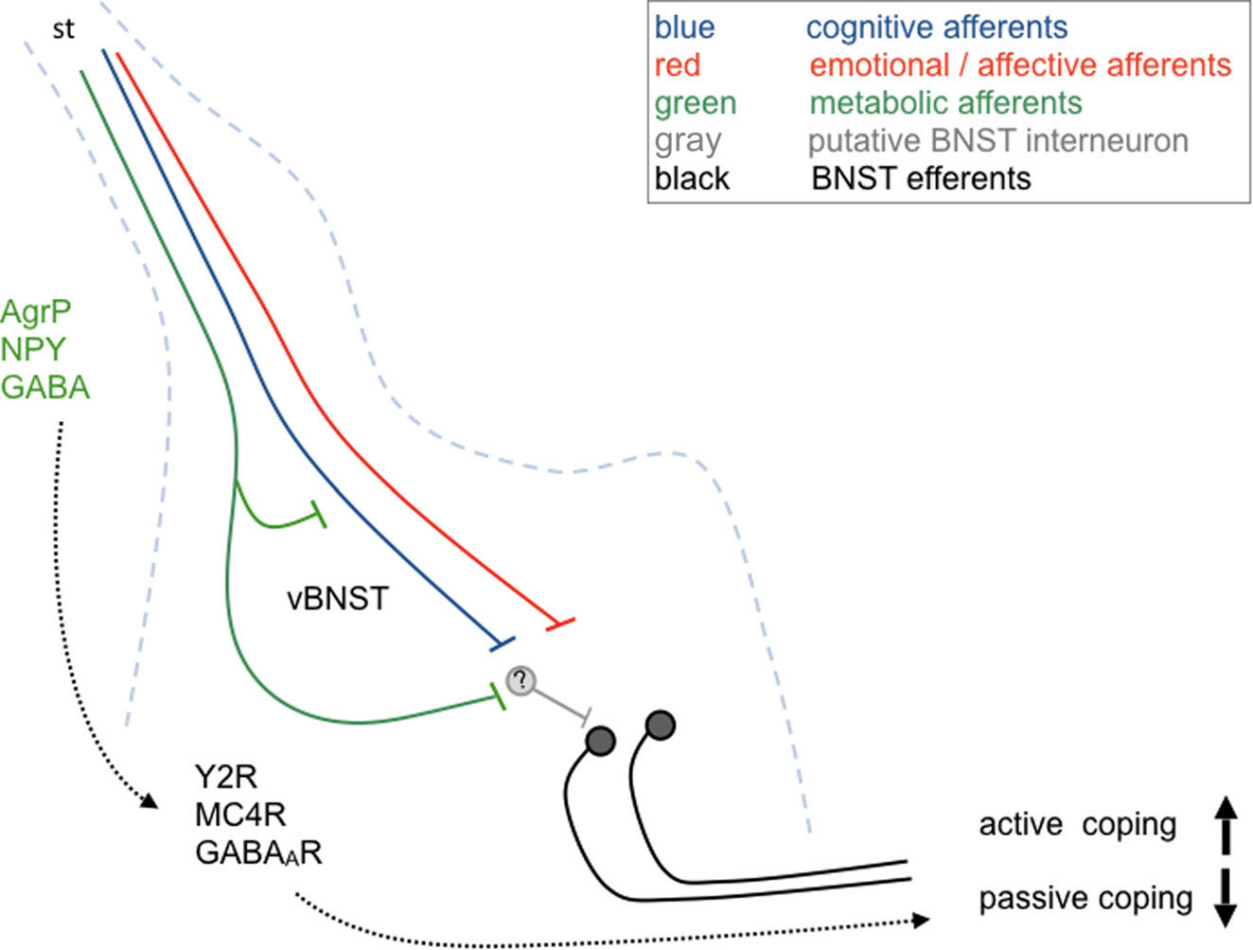
Proposed effect of agouti‐related peptide/neuropeptide Y (AgRP/NPY) axons in the ventral aspect of the bed nucleus of the stria terminalis (vBNST). Hunger activates AgRP/NPY neurons, which inhibit BNST afferents from different sources (cognitive, emotional‐affective) by activation of presynaptic Y2 receptor (Y2R). In addition, specific BNST subpopulations are inhibited by the activation of Y2R and GABA_A_ receptors (GABA_A_R) and by inhibition of melanocortin receptor (MC4R). Upon integration with cognitive and emotional‐affective afferents, the BNST may initiate a shift from passive to active stress coping, a phenomenon that would facilitate food seeking and promote survival even in the presence of potential threats.

The BNST is located in the ventral forebrain and is neurochemically, anatomically, and developmentally highly similar to the CeA. Most importantly, the CeA and BNST exhibit a similar distribution and content of modifying neuropeptides. The integrative role of the BNST as a valence center^165^ may induce a rapid shift from homeostasis to attention and threat encounter. As such, the BNST has to process various converging pieces of information about mood, motivation, energy supply, and external or internal threats in order to maintain an adaptive behavioral response. Indeed, anatomical tract tracing suggests intense, in part reciprocal, connectivity with several homeostatic and emotional‐affective brain areas.^166^ In this context, it is interesting to note that arcuate POMC neurons, which are involved in the chronic suppression of food intake, project to the BNST, which mediates sustained fear, while POMC neurons of the NTS, which inhibit food intake upon acute stimulation, connect preferentially to the CeA, which is responsible for short‐duration phasic fear.^81^, ^82^ Thus, different POMC neuronal populations may link chronic suppression of food intake to sustained avoidance, while acute suppression of food intake may be accompanied by phasic threat responses. On the other hand, AgRP/NPY neurons also densely innervate the vBNST, a subdivision that receives input from various fear and anxiety‐related structures^167^ (Figs. 2, 5, and 7). This includes the main amygdala nuclei, but also PVT, PAG, PBN, and serotonergic raphe, all brain areas that are targeted by arcuate AgRP/NPY and POMC neurons alike. Thus, the vBNST may constitute a central hub that integrates feeding and fear‐related processing on longer time scales, with influence from glutamatergic, GABAergic, and monoaminergic fibers, many of which contain modulatory neuropeptide transmitters. For instance, the vBNST has the strongest noradrenergic inputs in the brain,^168^ which often corelease NPY, and it receives dense innervation from hypothalamic, brainstem, and cortical areas, such as the insula, mPFC, and BLA. It may thus coordinate valence surveillance, which integrates stimuli of different modalities and priorities to generate a specific purposive behavior.^165^ For instance, stimulation of beta‐adrenoreceptors in the ventral BNST has been shown to reduce food intake while increasing anxiety.^169^ On the other hand, recent evidence demonstrates that NPY acting on vBNST Y2R reduces fear expression and promotes fear extinction.^63^, ^64^ Interestingly, the activation of vBNST Y2R was able to induce long‐term suppression of conditioned fear even in the absence of extinction training. Whether these Y2Rs are regionally expressed, or located presynaptically on axon terminals derived from various neuronal inputs, such as CeA^152^ or PVT,^167^ remains to be demonstrated. Furthermore, NPY, which ultimately activates these neurons, may be released from BNST neurons or derive from other sources, such as arcuate projections. Thus, during hunger, NPY originating from arcuate AgRP/NPY neurons may not only increase food intake, but also suppress anxiety‐ and fear‐related behavior by Y2R‐mediated suppression of specific BNST afferents/efferents. It was proposed that vGlut2‐containing vBNST projections to the VTA are anxiogenic and aversive, while vGAT‐containing vBNST neurons produce an anxiolytic and rewarding phenotype.^17^ However, it remains to be demonstrated whether AgRP/NPY neurons can reduce anxiety by the activation of Y2Rs located on vGlut2 BNST neurons (Fig. 7). It is equally conceivable that during states of nutritional depletion, activation of incoming AgRP/NPY fibers inhibits vGAT neurons of the vBNST, producing an intense state of aversion that favors active coping and search for food.^28^ Further studies are needed to identify the Y2R content of diverse neuronal subpopulations and their projection targets.

## Closing the loop––regulation of the PVH and HPA axis

The vBNST seems to be an intermediary source for PVH afferents preselecting and actively integrating converging modular stimuli from diverse higher order centers, such as PVT, hippocampus, and mPFC.^170^ Active and passive coping strategies correlate with PVH activity and often reflect susceptibility to stress‐related disease development, with passive approaches bearing higher risk.^171^ Activation of AgRP/NPY neurons may directly modulate PVH excitability, or indirectly by mostly inhibitory avBNST neurons. AgRP/NPY neurons send dense projections to the avBNST, and, in turn, those avBNST neurons projecting to the PVH receive inputs from higher order centers, including the hippocampus and mPFC.^170^ Thus, by this indirect modulation via the avBNST, AgRP/NPY neurons are able to integrate metabolic needs with corresponding emotional and cognitive processes.

The PVH is not only a direct projection target where orexigenic and anorexigenic homeostatic signaling converges, but also a major gateway to the periphery by synthesis of hypothalamic releasing factors targeting pituitary lobes. Furthermore, the PVH receives afferents from limbic brain areas to integrate emotional stimuli with respective hormonal responses. Hunger promotes fear extinction^2^, ^103^, ^163^ and the HPA axis is a possible gateway linking hunger and fear. Peripherally released ghrelin activates central arcuate AgRP/NPY neurons and NPY may provide a link to the activation of PVH CRH neurons, triggering the activation of the HPA axis.^99^, ^172^ NPY infusion into the ventricles^173^ or PVH results in the activation of CRH neurons and increases plasma corticosterone levels.^172^ However, since AgRP/NPY projections are mostly inhibitory in nature and Y1Rs are expressed by parvocellular neurons, while CRH neurons populate the magnocellular compartment, a local inhibitory intermediate may be involved.^174^ Thus, tonic inhibition of CRH secretagogues may be relieved during hunger by AgRP/NPY neurons, initiating stress hormone release and promote food seeking. Interestingly, recent evidence suggests a rapid decrease in CRH neuron activity in the PVH following sensory detection of food.^175^ This guarantees that as soon as a feeding source has been discovered, searching for food rapidly ceases and food intake commences. Multiple lines of evidence suggest that acute CRH release may promote active coping strategies and support food seeking directly or indirectly, but highly related to the activity of arcuate AgRP/NPY neurons. This may include CRH in CeL neurons^161^ or in PVH secretagogues.^172^, ^173^

## Limitations

When investigating emotional‐affective processes in rodents, conditioned freezing and approach‐avoidance responses are generally taken as measures for fear and anxiety‐related behaviors, respectively. However, fasting could result in hunger‐induced hyperactivity/arousal, which may be dependent or independent of fear and anxiety. This opens up the possibility of misinterpreting hyperlocomotion as absence of fear/anxiety. Thus, it is important to mention that fasting‐induced facilitation of fear extinction learning was also maintained when the same mice were tested for extinction memory recall under fed conditions. While beyond the scope of this review, several neuropeptides that predominantly increase food intake may induce generalized states of stress or even panic attacks.^176^ Furthermore, hypoglycemic arousal, which is frequently accompanied by sleep disturbances and increased locomotion,^177^, ^178^ may be mistaken by reduced fear or anxiety.

## Conclusions

It has been long known that fear and anxiety can reduce appetite; however, recent evidence demonstrates that the opposite is also true, that hunger can suppress fear and anxiety. An individual encounters continuous challenges and has to adapt purposive behaviors according to the most urgent needs to ensure survival. It appears that evolutionarily conserved neuropeptides are central elements controlling homeostatic balance and related emotional‐affective behaviors. Several lines of evidence suggest that those neuropeptides, which are increased by hunger (NPY and AgRP), also reduce fear and anxiety, while others released upon refeeding and in particular during states of satiety are predominantly anxiogenic (α‐MSH, CRH, and CCK). This makes sense when considering that a hungry individual has to make a choice between staying in a safe shelter and starving, or to encounter potentially dangerous environments in search of food and survival. However, an indiscriminate reduction of fear and anxiety would be an unacceptable risk. Hence, acute release of stress hormones may protect the individual by increasing arousal and alertness, while their long‐term increase could lead to pathology. Although large data sets from various laboratories support this hypothesis, much of the details, in particular regarding microcircuits and their mutual interaction, are still missing and remain speculative. Regardless of this, disentangling survival circuits and identifying the involved interaction molecules will eventually open the door for novel treatment strategies addressing metabolic and emotional‐affective disorders.

## Competing interests

The authors declare no competing interests.
